# ROP64-Deficient *Toxoplasma gondii* Strain Confers Protection Against Acute and Chronic Toxoplasmosis in Mice

**DOI:** 10.3390/microorganisms14081725

**Published:** 2026-08-06

**Authors:** Zhi Zheng, Shi-Bo Huang, Yi-Rong Ma, Wen-Bo Hao, Xiao-Nan Zheng, Hong-Yu Song, Xing-Quan Zhu

**Affiliations:** 1 Shanxi Key Laboratory of Animal Disease Research, Prevention and Control, College of Veterinary Medicine, Shanxi Agricultural University, Jinzhong 030801, China; 15863201790@163.com (Z.Z.); h17516211643@126.com (S.-B.H.); mayirong09@163.com (Y.-R.M.); 16696739873@163.com (W.-B.H.); zhengxiaonan8889@126.com (X.-N.Z.); 2 School of Basic Medical Sciences, Changzhi Medical College, Changzhi 046000, China

**Keywords:** *Toxoplasma gondii*, ROP64, live attenuated vaccine, immune response, virulence attenuation, cyst burden

## Abstract

Toxoplasmosis, caused by *Toxoplasma gondii*, remains an important zoonotic disease affecting both human and animal health, particularly in immunocompromised individuals and during pregnancy. Current treatments are unable to eliminate latent tissue cysts and are further limited by adverse effects and prolonged treatment regimens, highlighting the need for effective vaccines. In this study, we evaluated the virulence attenuation and protective efficacy of a CRISPR–Cas9-generated rhoptry protein 64 knockout strain (RHΔ*rop64*) as a live attenuated vaccine candidate in Kunming mice. RHΔ*rop64* exhibited markedly reduced virulence, and mice tolerated doses of up to 1 × 10^2^ tachyzoites without obvious clinical signs. Immunization provided substantial protection against acute challenge with the parental RHΔ*ku80* and ToxoDB#9 (PYS) strains at 30 days post-vaccination and partial protection against type II (Pru) tachyzoites. In addition, all immunized mice survived following oral challenge with 10 or 40 Pru cysts and exhibited reduced brain cyst burdens among surviving mice. Vaccination also induced measurable humoral and cytokine responses, characterized by increased levels of *T. gondii*-specific IgG and cytokines that were maintained for at least 75 days post-vaccination. These results suggest that RHΔ*rop64* has the potential to serve as a live attenuated vaccine candidate, providing substantial, although incomplete, protection against acute challenge with the three *T. gondii* strains tested and being associated with reduced brain cyst burdens among surviving mice following chronic challenge. Further evaluation is required to determine its safety and applicability as a vaccine candidate for food-producing animals and cats.

## 1. Introduction

Toxoplasmosis is a globally prevalent parasitic zoonosis caused by *Toxoplasma gondii* which can infect almost all warm-blooded animals and humans. Domestic and wild felines serve as definitive hosts, shedding unsporulated oocysts into the environment via feces [1]. Transmission to intermediate hosts, including humans, primarily occurs through ingestion of tissue cysts in undercooked meat or oocysts in contaminated food and water [1,2]. In immunocompetent individuals, infection is typically asymptomatic or subclinical. However, in immunocompromised individuals—such as those undergoing chemotherapy, organ transplantation, or those with acquired immunodeficiency syndrome (HIV/AIDS)—latent tissue cysts can reactivate, leading to severe or even life-threatening disease [3,4].

Following acute infection, rapidly replicating tachyzoites differentiate into slowly replicating bradyzoites and form tissue cysts, predominantly within the brain and muscle tissues. These tissue cysts can persist throughout the host’s lifetime and represent a major source of recrudescent disease in immunocompromised individuals. Importantly, tissue cysts are largely refractory to currently available therapies, making chronic infection one of the major challenges in the treatment of toxoplasmosis [1,2].

In addition to its complex life cycle and ability to establish chronic infection, *T. gondii* exhibits substantial genetic diversity. Although it is commonly classified into three major clonal lineages (types I, II, and III) in North America and Europe, numerous atypical strains have also been identified, particularly in South America, further highlighting the extensive genetic diversity of this parasite. Representative strains of the three major clonal lineages include RH and GT1 (type I), Pru and ME49 (type II), and VEG and CTG (type III) [5,6,7]. Notably, strains differ markedly in virulence, with type I strains being highly virulent in mice, whereas type II and III strains are comparatively less virulent and are often associated with chronic infection.

Current treatment of toxoplasmosis mainly relies on antifolate drugs such as py-rimethamine combined with sulfonamides, which are effective against the acute stage but unable to eliminate latent tissue cysts [8,9]. Moreover, the long treatment duration and potential adverse effects limit clinical applicability [10]. These challenges, together with the parasite’s ability to establish chronic infection and evade host immune responses, highlight the urgent need for effective preventive strategies. In this context, vaccine development has attracted increasing attention. Various vaccine strategies, including inactivated, live attenuated, DNA, protein, epitope, and vector-based vaccines, have been extensively evaluated in animal models [11,12,13]. Among these, live attenuated vaccines are considered particularly promising, as they mimic natural infection and induce strong and long-lasting protective immunity [12,14,15].

Recent advances in CRISPR–Cas9 genome editing have facilitated the rational de-sign of genetically attenuated *T. gondii* strains with potential as live vaccines. For example, gene-disrupted RH strains targeting factors such as putative transporter 1 or GRA17 have been shown to provide protection against both homologous and heterologous challenges [16,17]. Similarly, engineered strains including WH3Δ*rop18* and PruΔ*rop38* exhibit reduced virulence and can provide varying degrees of protection against acute and chronic infection [18,19]. These studies underscore the feasibility of developing live attenuated vaccines through targeted genetic manipulation of virulence-associated genes.

Rhoptries, which are unique to apicomplexan parasites, are one of the three apical secretory organelles in *T. gondii*, together with dense granules and micronemes [20,21]. Structurally composed of a neck and a bulb region, rhoptries play essential roles during host cell invasion and intracellular survival [20,21,22,23]. Rhoptry proteins (ROPs) function as key effectors involved in parasitophorous vacuole (PV) formation, host cell manipulation, and immune modulation [24]. Large-scale proteomic and spatial localization studies have identified a diverse repertoire of ROPs in *T. gondii*, many of which remain functionally uncharacterized. Importantly, several ROPs have been implicated in parasite virulence and host immune regulation, making them attractive targets for vaccine development [25].

In our previous work, a previously uncharacterized putative rhoptry protein encoded by TGME49_279420 was endogenously tagged and confirmed to localize to rhoptries in both tachyzoite and bradyzoite stages, and was subsequently designated as ROP64 [26]. Its localization in both invasive and chronic developmental stages suggested that ROP64 may play a role throughout the parasite life cycle. Functional characterization revealed that deletion of ROP64 did not affect parasite growth in vitro but significantly attenuated virulence in vivo in that mice infected with RHΔ*rop64* tachyzoites survived markedly longer than those infected with the parental RHΔ*ku80* strain, and transcriptomic analysis further suggested that ROP64 may participate in parasite–host interactions associated with immune evasion [26]. Given the established roles of rhoptry proteins in parasite virulence and host–parasite interactions, together with the marked attenuation observed following ROP64 deletion, we hypothesized that the attenuated RHΔ*rop64* strain may serve as a potential live attenuated vaccine candidate. Therefore, the present study aimed to evaluate its protective efficacy against both acute and chronic *T. gondii* infection in mice, as well as the associated humoral and cellular immune responses.

## 2. Materials and Methods

### 2.1. Mice and Parasites

Eight-week-old female Kunming mice were purchased from Beijing Sibeifu Biotechnology Co., Ltd. (Beijing, China). Animals were maintained under specific pathogen-free (SPF) conditions at 25 °C with 50–60% humidity and were provided with food and water ad libitum. All mice were acclimated for one week prior to experimentation. All procedures were performed in accordance with institutional guidelines for animal care and use, and efforts were made to minimize animal suffering.

The *T. gondii* type II Pru strain, the ToxoDB#9 PYS strain, parental RHΔ*ku80* strain (referred to as RH), and the knockout RHΔ*ku80*Δ*rop64* strain (referred to as RHΔ*rop64*) were used in this study. Throughout the manuscript, “RH” refers exclusively to the parental RHΔ*ku80* strain. The wild-type RH strain was not used in this study. The RHΔ*rop64* strain used in this study was generated and characterized previously using a CRISPR-Cas9-mediated gene disruption strategy in the RHΔ*ku80* background [26]. Parasites were maintained in human foreskin fibroblasts (HFFs) cultured in Dulbecco’s modified Eagle’s medium (DMEM; Gibco, Grand Island, NY, USA) supplemented with 2% fetal bovine serum (FBS) at 37 °C in 5% CO_2_. Tissue cysts of the Pru strain were obtained from brain homogenates of infected mice as previously described [27].

### 2.2. Optimization of the RHΔrop64 Vaccination Dose

To evaluate the in vivo virulence of the RHΔ*rop64* strain and determine the optimal immunization dose, groups of eight-week-old Kunming mice (*n* = 6 per group) were intraperitoneally injected with RHΔ*rop64* tachyzoites at doses of 1 × 10^2^, 1 × 10^3^, 1 × 10^4^, 1 × 10^5^, or 1 × 10^6^. A control group received 1 × 10^2^ RH tachyzoites. Mice were monitored twice daily for clinical signs and survival for up to 35 days post-infection (dpi).

### 2.3. Evaluation of Protection Against Acute and Chronic Toxoplasmosis

**Figure 1:**
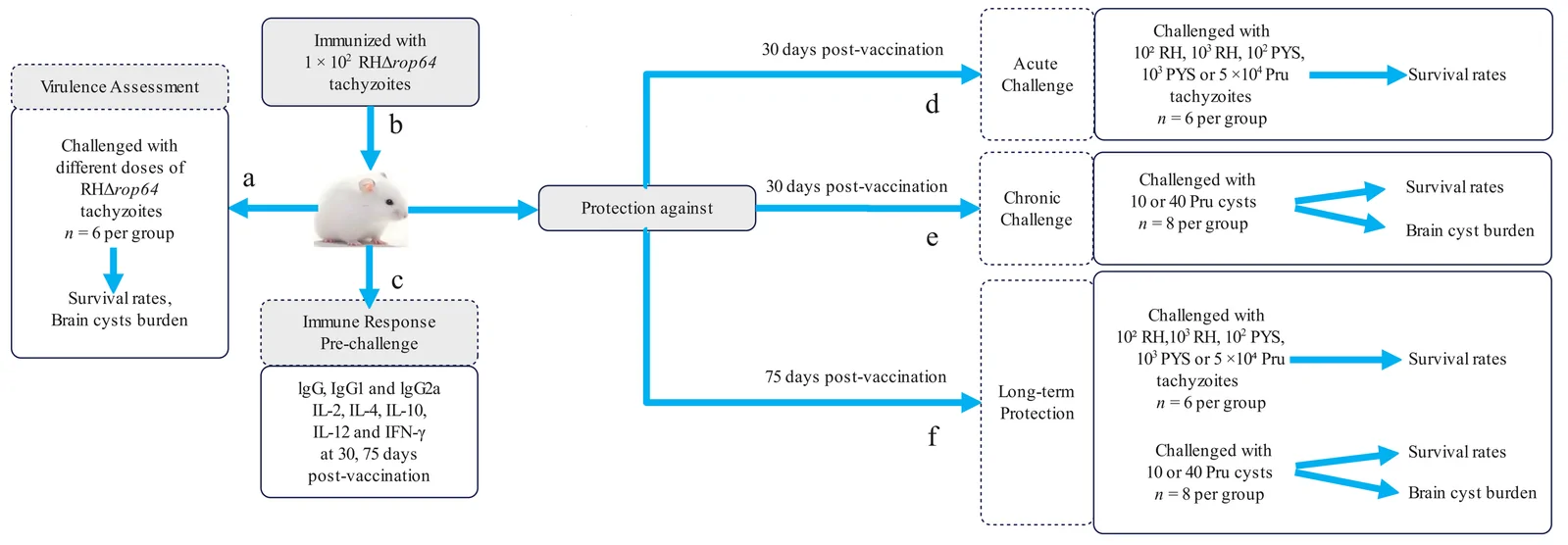
Experimental design of RHΔ*rop64* immunization and challenge assays. Schematic overview of the study design, including virulence assessment of RHΔ*rop64* in mice (**a**), immunization with RHΔ*rop64* tachyzoites (**b**), evaluation of humoral and cellular immune responses (**c**), and assessment of protective efficacy against acute (**d**), chronic (**e**), and long-term infection (**f**).

The overall experimental design of the immunization and challenge procedures is illustrated in Figure 1. Mice were immunized via intraperitoneal injection with 1 × 10^2^ RHΔ*rop64* tachyzoites or an equal volume of phosphate-buffered saline (PBS).

#### 2.3.1. Evaluation of Protection Against Acute Toxoplasmosis

To evaluate protection against acute toxoplasmosis, immunized and naïve mice (*n* = 6 per group) were challenged intraperitoneally at 30 and 75 days post-vaccination (dpv) with 1 × 10^2^ or 1 × 10^3^ tachyzoites of RH or PYS strains, or with 5 × 10^4^ tachyzoites of the Pru strain. Parasite viability and inoculum size were confirmed by plaque assay prior to challenge.

Challenge strains and doses were selected based on both parasite virulence and genetic background, following previously published vaccine evaluation studies [18,28]. RH (type I) and PYS (ToxoDB#9) strains are highly virulent in mice and were therefore administered at doses of 1 × 10^2^ or 1 × 10^3^ tachyzoites. In contrast, the less virulent type II Pru strain was administered at a higher dose (5 × 10^4^ tachyzoites) to establish a consistent challenge infection for evaluation of protective efficacy. In addition, RH, Pru, and PYS strains represent distinct genetic backgrounds and were included to evaluate protection induced by RHΔ*rop64* against the three *T. gondii* strains with distinct genetic backgrounds used in this study.

Mice were monitored daily for clinical signs and survival throughout the experiment. Survival analyses were based on natural mortality, and no animals were euthanized prior to death for endpoint determination.

#### 2.3.2. Evaluation of Protection Against Chronic Toxoplasmosis

To assess protection against chronic infection, immunized and naïve mice (*n* = 8 per group) were orally inoculated with 10 or 40 Pru cysts at 30 and 75 dpv. A slightly larger sample size was used because the oral cyst challenge model exhibits greater biological variability than the acute tachyzoite challenge model. For each challenge time point, an independent PBS-treated naïve group was included and challenged in parallel with the corresponding RHΔ*rop64*-immunized group. Clinical signs and survival were monitored for 35 dpi.

At 35 dpi, all surviving mice were euthanized, and brain cysts were enumerated microscopically. Brain cyst counts of zero (i.e., no microscopically detectable cysts) were included in the calculation of the mean brain cyst burden. Samples without microscopically detectable cysts were further analyzed by conventional PCR amplification targeting the *T. gondii B1* gene to assess the presence of parasite DNA [28]. Primer sequences, PCR reaction mixture and amplification conditions are provided in Supplementary File S1: Tables S1–S3.

### 2.4. Preparation of Soluble T. gondii Antigen (STAg)

Freshly collected tachyzoites were washed three times with pre-chilled PBS and resuspended in PBS. Parasites were lysed by repeated freeze–thaw cycles followed by sonication on ice (80 W, 30 min). The lysate was centrifuged at 12,000× *g* for 10 min, and the supernatant was harvested and stored at −80 °C until use.

### 2.5. Measurement of T. gondii-Specific Antibodies

Humoral immune responses were evaluated at 30 and 75 dpv by measuring total IgG and IgG subclasses using enzyme-linked immunosorbent assay (ELISA). Briefly, 96-well plates were coated with STAg (1 μg/well) at 37 °C for 2 h and incubated overnight at 4 °C. Plates were washed with PBST and blocked with 5% bovine serum albumin (BSA) at 37 °C for 1 h. Serum samples diluted 1:100 in 1% BSA were added and incubated at 37 °C for 2 h. Subsequently, horseradish peroxidase (HRP)-conjugated goat anti-mouse IgG (Abcam, Cambridge, UK) was used at a dilution of 1:3000, while HRP-conjugated goat anti-mouse IgG1 and IgG2a (Abcam, Cambridge, UK) were used at a dilution of 1:5000. The plates were incubated at 37 °C for 1 h and then washed. Subsequently, 3,3′,5,5′-tetramethylbenzidine (TMB) substrate solution (Beyotime Biotech, Shanghai, China) was added for color development. The reaction was stopped with 2% sulfuric acid, and optical density was examined at 450 nm.

### 2.6. Cytokine Detection in Splenocyte Cultures

For cytokine analysis, spleens were aseptically collected from immunized and PBS-treated naïve mice at 30 and 75 dpv, before challenge infection, as previously described [29]. Single-cell suspensions were prepared by mechanical disruption through a 200-mesh nylon filter. Cells were centrifuged, and erythrocytes were lysed using red blood cell lysis buffer. After washing, cells were resuspended in RPMI 1640 medium supplemented with 10% FBS. Cell viability was assessed by trypan blue exclusion, and samples with >95% viability were used. Splenocytes were adjusted to 3 × 10^6^ cells/mL and stimulated with STAg (10 μg/mL). Culture supernatants were collected at specific time points: IL-2 and IL-4 at 24 h, IL-10 at 72 h, and IL-12 and IFN-γ at 96 h. Cytokine levels were examined using ELISA kits (BioLegend, San Diego, CA, USA) following the manufacturer’s instructions.

### 2.7. Statistical Analysis

All experiments were conducted with at least three independent biological replicates. Statistical analyses were performed using GraphPad Prism 8.0 (GraphPad Software, Inc., La Jolla, CA, USA). Differences between two groups were analyzed using unpaired two-tailed Welch’s *t*-tests. Comparisons of brain cyst burdens involving three groups were performed using one-way analysis of variance (ANOVA) followed by Dunnett’s multiple-comparison test. Survival curves were compared using the Mantel–Cox log-rank test. A *p* value < 0.05 was considered statistically significant, with *p* < 0.01, <0.001, and <0.0001 representing different levels of significance.

## 3. Results

### 3.1. Attenuation of Virulence and Optimization of Vaccination Dose

Deletion of the ROP64 gene markedly attenuated the virulence of *T. gondii*. To evaluate both virulence and the suitability of RHΔ*rop64* as a live attenuated vaccine, Kunming mice were intraperitoneally injected with different doses of RHΔ*rop64* tachyzoites.

**Figure 2:**
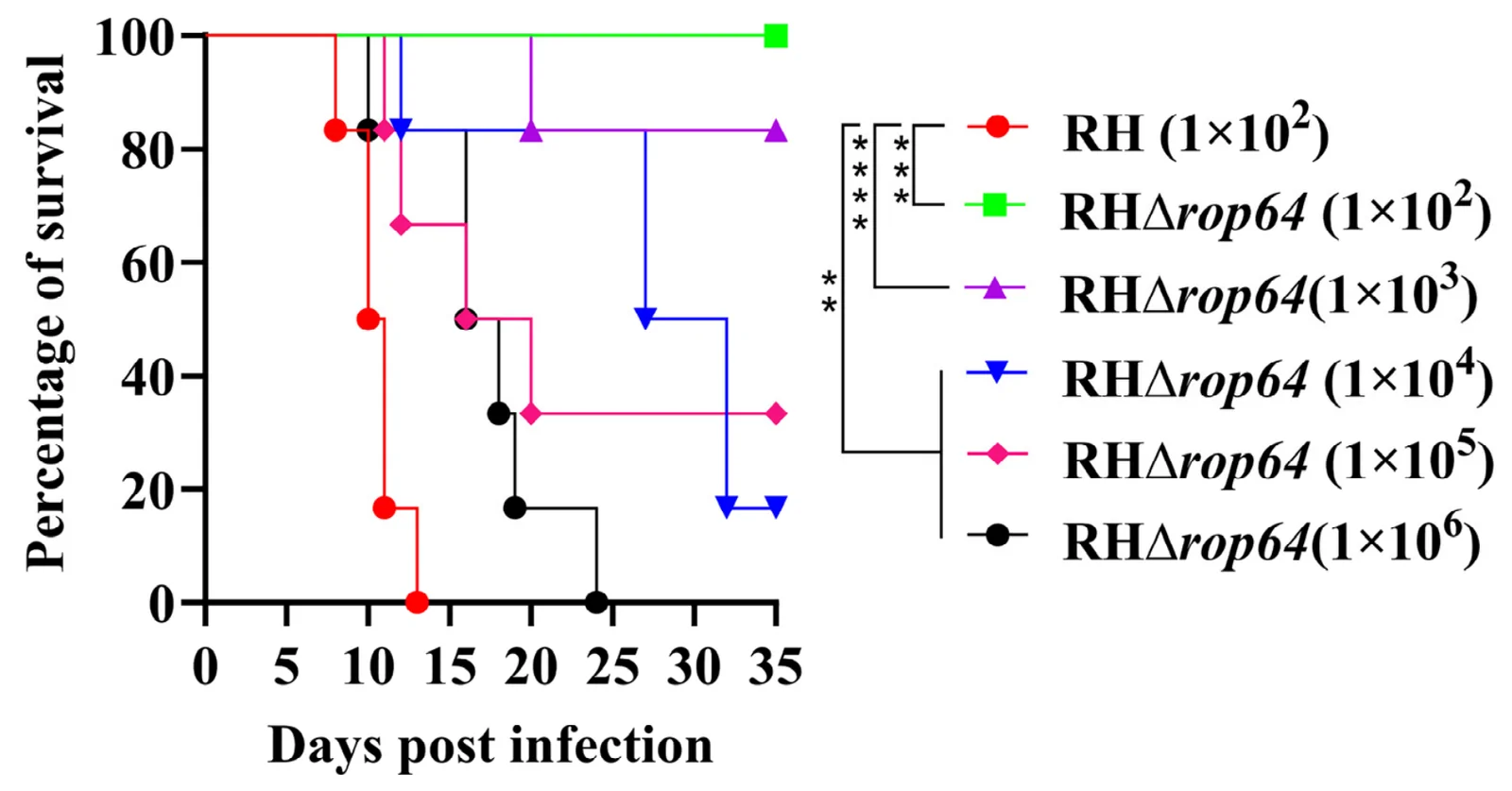
Attenuation of virulence of the RHΔ*rop64* strain in mice. Survival curves of Kunming mice intraperitoneally infected with increasing doses (1 × 10^2^, 1 × 10^3^, 1 × 10^4^, 1 × 10^5^, and 1 × 10^6^) of RHΔ*rop64* tachyzoites or 1 × 10^2^ RH tachyzoites. Statistical significance was determined by the log-rank (Mantel–Cox) test. **** *p* < 0.0001, *** *p* < 0.001, ** *p* < 0.01.

All mice infected with 1 × 10^2^ RHΔ*rop64* tachyzoites survived. Mice infected with 1 × 10^3^ tachyzoites showed an 83.33% survival rate, and prolonged survival was observed even at the highest dose of 1 × 10^6^. In contrast, all mice infected with 1 × 10^2^ RH tachyzoites succumbed to infection (Figure 2).

Mice infected with RHΔ*rop64* exhibited only mild clinical signs, characterized primarily by slightly ruffled fur at the highest dose. Notably, mortality occurred relatively late during infection. In contrast, mice infected with the parental RHΔ*ku80* strain developed severe clinical symptoms, including ruffled fur, reduced activity, and decreased food intake, and succumbed rapidly to infection.

These results demonstrate substantial attenuation of virulence in RHΔ*rop64*. Based on these findings, a dose of 1 × 10^2^ tachyzoites was selected for subsequent immunization experiments.

### 3.2. RHΔrop64 Confers Protection Against Acute and Chronic Toxoplasmosis

**Figure 3:**
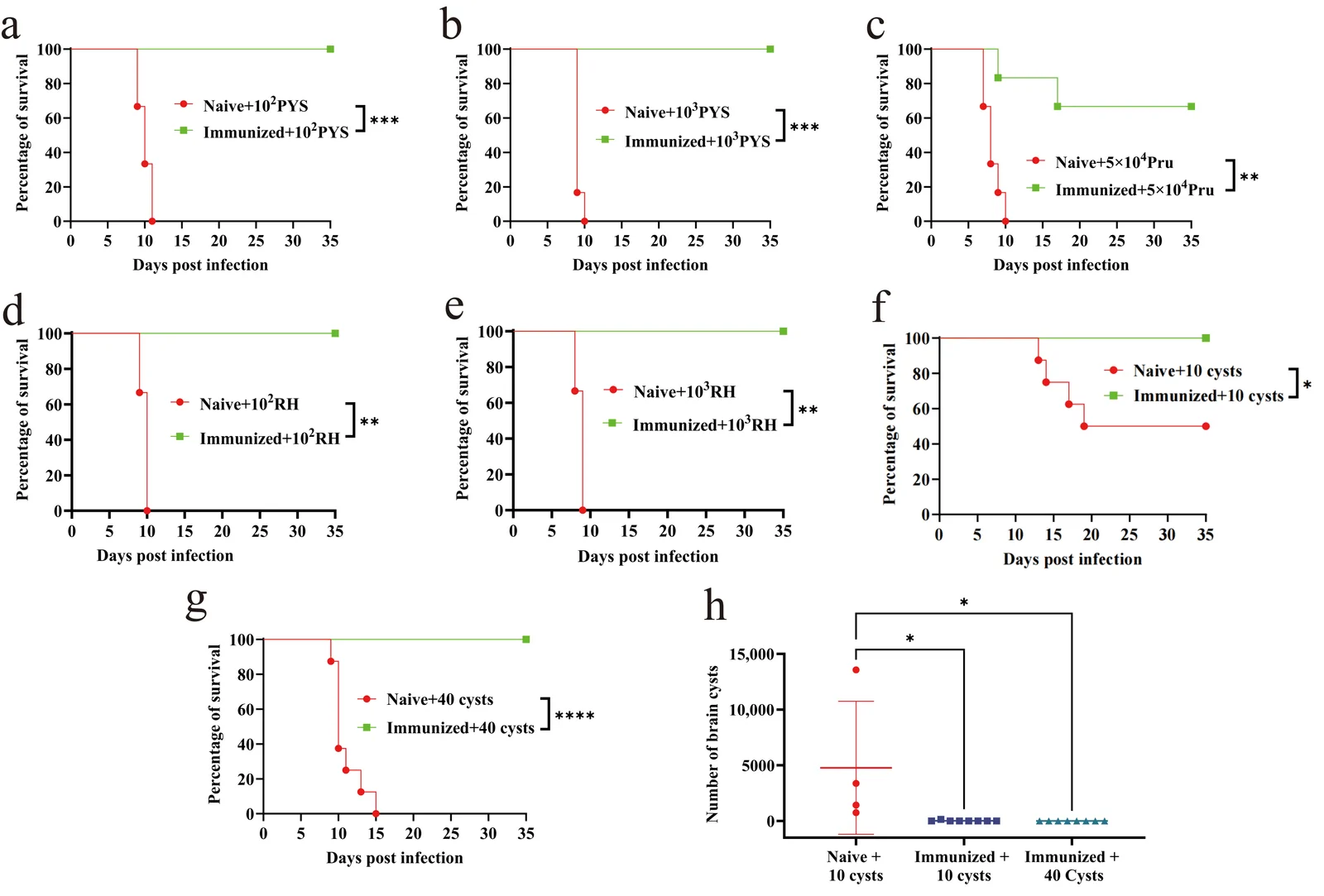
Protective efficacy of RHΔ*rop64* vaccination against acute and chronic *Toxoplasmosis* at 30 dpv. Mice immunized with 1 × 10^2^ RHΔ*rop64* tachyzoites were challenged with tachyzoites of different genotypes, including 1 × 10^2^ PYS (**a**), 1 × 10^3^ PYS (**b**), 5 × 10^4^ Pru (**c**), 1 × 10^2^ RH (**d**), and 1 × 10^3^ RH (**e**). Survival was monitored for 35 days (*n* = 6 per group). For chronic infection, mice were orally challenged with 10 or 40 Pru cysts (**f**,**g**), and survival was recorded for 35 days (*n* = 8 per group). Brain cyst burden in surviving mice was quantified at 35 dpi (**h**). Statistical significance: **** *p* < 0.0001, *** *p* < 0.001, ** *p* < 0.01, * *p* < 0.05.

To evaluate the protective efficacy of RHΔ*rop64* vaccination, immunized and naïve mice were challenged with tachyzoites of different *T. gondii* or with Pru cysts at 30 dpv (Figure 3).

Following challenge with type I (RH) and ToxoDB#9 (PYS) strains, all immunized mice survived, whereas all naïve mice died within 11 days (Figure 3a,b,d,e). After challenge with type II Pru tachyzoites, RHΔ*rop64*-immunized mice exhibited a survival rate of 66.67% (Figure 3c). In addition, among surviving immunized mice, the mean brain cyst burden was 19 ± 38, and only 1 of 4 mice had microscopically detectable brain cysts (Supplementary File S2: Table S4). PCR analysis of brain tissues without microscopically detectable cysts detected the *T. gondii B1* gene in 3 of 4 surviving mice (Supplementary File S2: Table S4; Supplementary File S3: Figure S1).

To assess protection against chronic infection, immunized and naïve mice were orally challenged with 10 or 40 Pru cysts at 30 dpv. All immunized mice survived both challenge conditions. In contrast, naïve mice began to show clinical signs at 8 dpi, with mortality starting at 9 dpi. Survival rates in naïve groups were 50% (low dose) and 0% (high dose) (Figure 3f,g).

Brain cyst burden was further evaluated in surviving mice at 35 dpi. Because cyst quantification was performed only in surviving animals, brain cyst burden could not be determined in the naïve group challenged with 40 cysts at 30 dpv, as no mice survived to the scheduled sampling time point. Among groups in which surviving naïve mice were available, immunized mice exhibited substantially lower brain cyst burdens and a lower proportion of mice with microscopically detectable brain cysts. Following challenge with 10 cysts, immunized mice harbored only 19 ± 53 cysts on average, whereas no microscopically detectable brain cysts were observed after challenge with 40 cysts (Figure 3h). PCR analysis was performed only in brain tissues from surviving mice without microscopically detectable brain cysts. Among these samples, the *T. gondii B1* gene was detected in 8 mice challenged with either 10 or 40 cysts (Supplementary File S2: Table S4; Supplementary File S3: Figure S1). These results indicate the presence of parasite DNA in some animals but do not provide information regarding parasite viability.

Overall, these results indicate that RHΔ*rop64* vaccination provides substantial protection against both acute and chronic *T. gondii* challenge and is associated with reduced brain cyst burdens among surviving mice.

### 3.3. Long-Term Protection Against Acute and Chronic Toxoplasmosis

**Figure 4:**
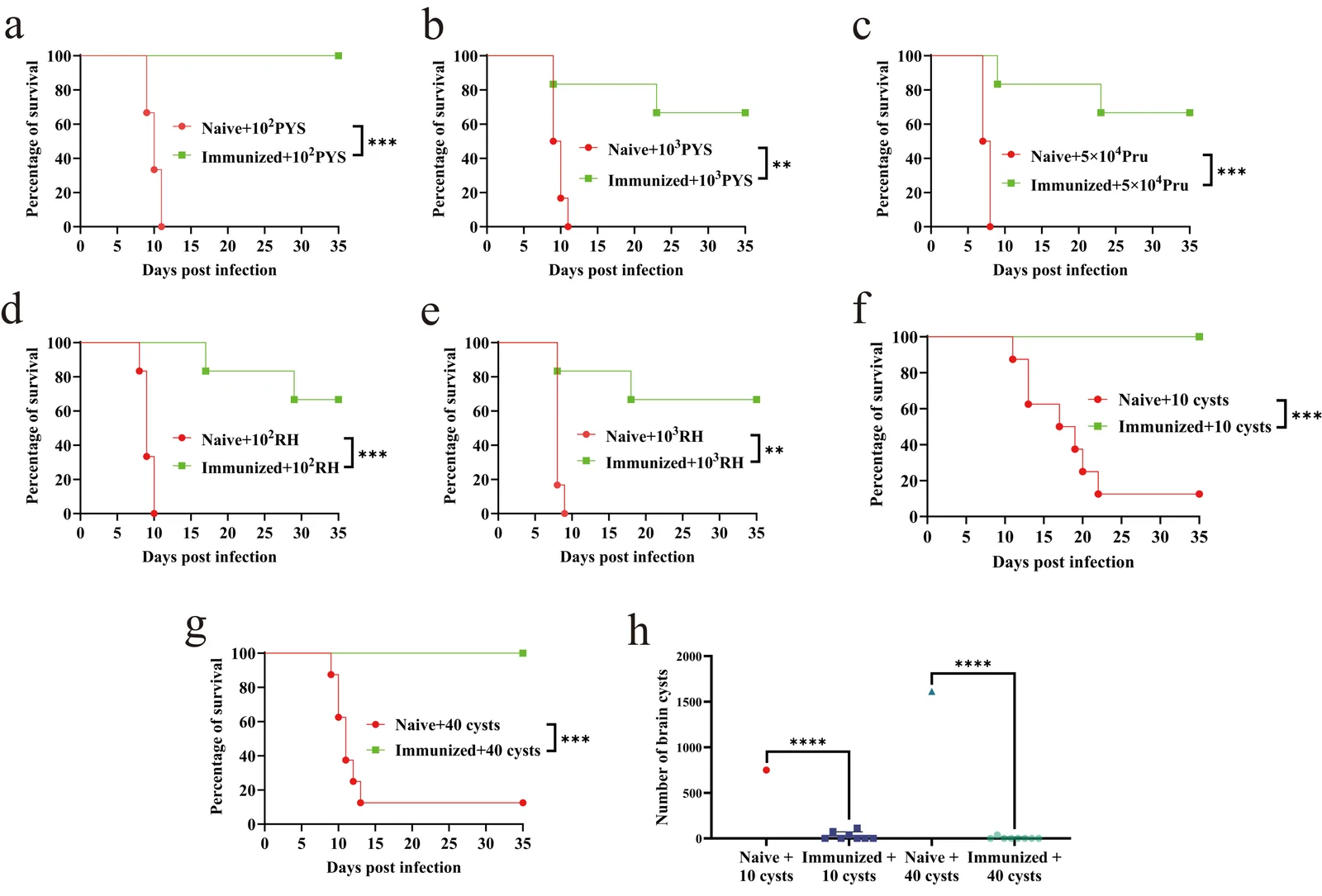
Long-term protective efficacy of RHΔ*rop64* vaccination against acute and chronic toxoplasmosis at 75 dpv. Mice were challenged with tachyzoites of different genotypes, including 1 × 10^2^ PYS (**a**), 1 × 10^3^ PYS (**b**), 5 × 10^4^ Pru (**c**), 1 × 10^2^ RH (**d**), and 1 × 10^3^ RH (**e**). Survival was monitored for 35 days (*n* = 6 per group). For chronic infection, mice were orally challenged with 10 or 40 Pru cysts (**f**,**g**), and survival was recorded for 35 days (*n* = 8 per group). Brain cyst burden in immunized and naïve mice was quantified at 35 dpi (**h**). Statistical significance: **** *p* < 0.0001, *** *p* < 0.001, ** *p* < 0.01.

To evaluate long-term protection, mice were challenged at 75 dpv with different *T. gondii* strains (Figure 4). Immunized mice exhibited a survival rate of 100% following challenge with 1 × 10^2^ PYS tachyzoites (Figure 4a). Survival rates of 66.67% were observed following challenge with 1 × 10^3^ PYS or 5 × 10^4^ Pru tachyzoites (Figure 4b,c). Following challenge with the highly virulent RH strain, immunized mice showed a survival rate of 66.67% regardless of whether 1 × 10^2^ or 1 × 10^3^ tachyzoites were administered (Figure 4d,e). In contrast, all naïve mice succumbed to infection following challenge with PYS, Pru, or RH strains.

For chronic infection, immunized mice maintained 100% survival after oral challenge with 10 or 40 Pru cysts at 75 dpv, whereas naïve mice showed survival rates of only 12.5% in both groups (Figure 4f,g). Among surviving mice, immunized mice exhibited both lower brain cyst burdens and a lower proportion of mice with microscopically detectable brain cysts than surviving naïve mice. In surviving naïve mice, mean cyst counts reached 750 and 1613 following challenge with 10 and 40 cysts, respectively, whereas immunized mice harbored substantially fewer cysts (28 ± 44 and 5 ± 13, respectively) (Figure 4h). PCR analysis of brain tissues without microscopically detectable brain cysts further confirmed the presence of the *T. gondii B1* gene in 9 surviving immunized mice (Supplementary File S2: Table S4; Supplementary File S3: Figure S1). These findings indicate the presence of parasite DNA in some animals but should be interpreted cautiously because PCR does not distinguish between viable and non-viable parasites.

These results indicate that RHΔ*rop64* vaccination confers sustained protective efficacy, particularly against chronic infection, although protection against highly virulent strains remains incomplete.

### 3.4. Humoral and Cellular Immune Responses Elicited by RHΔrop64 Immunization

**Figure 5:**
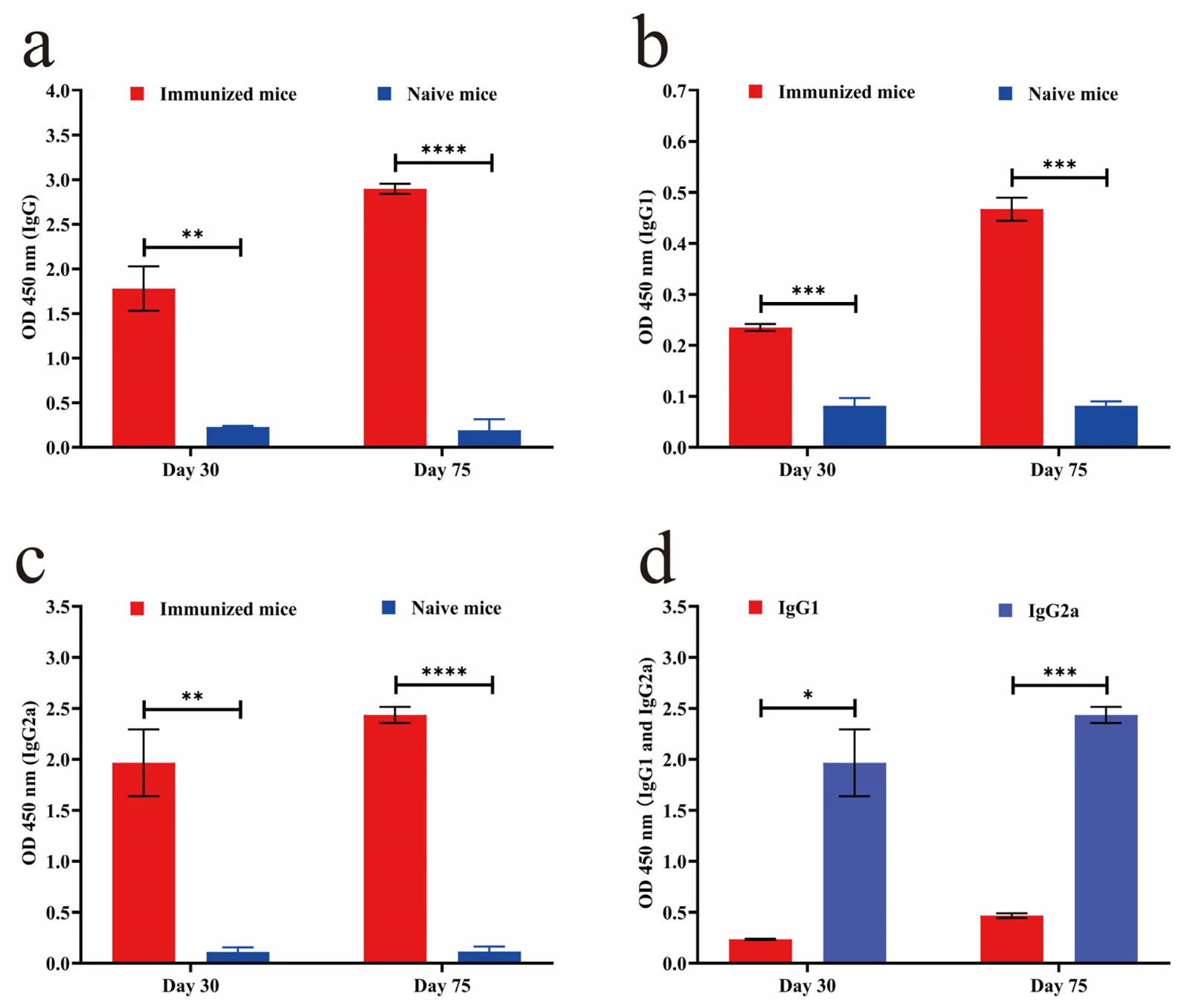
Humoral immune responses elicited by RHΔ*rop64* immunization. Levels of total IgG (**a**), IgG1 (**b**), and IgG2a (**c**) in serum collected from RHΔ*rop64*-immunized and PBS-treated naïve mice at 30 and 75 dpv. Comparison of IgG1 and IgG2a levels (**d**). In panels (**a**–**c**), colors indicate experimental groups (immunized vs. naïve), whereas in panel (**d**), colors indicate antibody subclasses (IgG1 vs. IgG2a). Statistical significance: **** *p* < 0.0001, *** *p* < 0.001, ** *p* < 0.01, * *p* < 0.05.

To assess humoral immunity, serum samples were collected at 30 and 75 dpv. Immunized mice showed significantly higher levels of *T. gondii*-specific total IgG, IgG1, and IgG2a compared with naïve mice (Figure 5). IgG2a levels were consistently higher than IgG1, suggesting an antibody profile consistent with a Th1-biased immune response.

**Figure 6:**
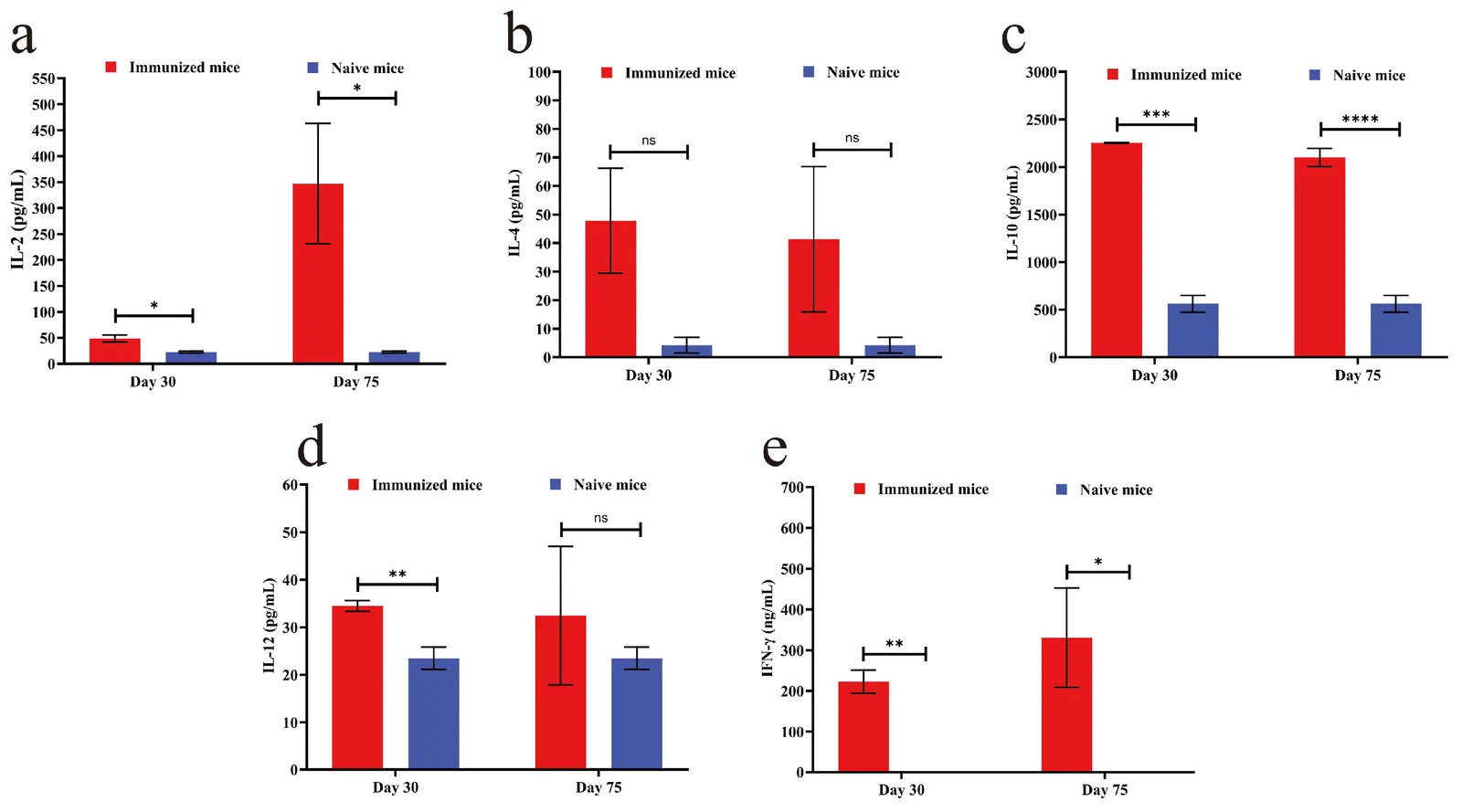
Cytokine responses elicited by RHΔ*rop64* immunization. Cytokine production in STAg-stimulated splenocyte cultures from immunized and PBS-treated naïve mice at 30 and 75 dpv, including IL-2 (**a**), IL-4 (**b**), IL-10 (**c**), IL-12 (**d**), and IFN-γ (**e**). Statistical significance: **** *p* < 0.0001, *** *p* < 0.001, ** *p* < 0.01, * *p* < 0.05; ns, not significant.

Cellular immune responses elicited by RHΔ*rop64* immunization were evaluated by measuring cytokine production in STAg-stimulated splenocyte cultures. Compared with PBS-treated naïve mice, RHΔ*rop64*-immunized mice exhibited increased production of Th1-associated cytokines (IL-2, IL-12, and IFN-γ) as well as Th2-associated cytokines (IL-4 and IL-10), with IFN-γ showing the most pronounced increase (Figure 6).

Overall, these results indicate that RHΔ*rop64* immunization elicits marked antigen-specific antibody and cytokine responses, characterized by strong antigen-specific antibody production and cytokine secretion.

## 4. Discussion

Toxoplasmosis remains a considerable issue of global public health, specifically for immunocompromised individuals and pregnant women [3]. Current treatments, such as pyrimethamine combined with sulfadiazine, are effective against acute infection but fail to eliminate latent tissue cysts and are further limited by adverse effects and prolonged treatment regimens [9]. These limitations, together with the complex life cycle and immune evasion capacity of *T. gondii*, highlight the urgent need for effective vaccines [15]. However, no commercial vaccines are currently available for humans, and vaccine candidates for most livestock have yet to achieve widespread application [14,30].

Existing vaccine strategies have shown varying degrees of efficacy but also present notable limitations. DNA and protein vaccines can induce Th1-type immune responses but often provide incomplete protection, possibly due to limited antigenic coverage [11,12]. Similarly, live attenuated strains derived from highly virulent backgrounds have demonstrated protective potential but still exhibit important limitations, including incomplete attenuation, suboptimal immune protection, or insufficient reduction in brain cyst burden [18,31,32]. These challenges underscore the need for improved vaccine candidates capable of inducing broader and more durable protection.

Several CRISPR/Cas9-generated live-attenuated *T. gondii* vaccine strains, including Δ*rop18*, Δ*rop38*, Δ*gra17*, Δ*gra72*, and Δ*cdpk3*, have recently demonstrated encouraging protective efficacy in murine models [16,17,18,19,28,33]. Similar to these vaccine candidates, RHΔ*rop64* conferred protection against both acute and chronic toxoplasmosis, markedly reduced brain cyst burdens, and elicited robust parasite-specific humoral and cellular immune responses. Notably, immunization with 1 × 10^2^ RHΔ*rop64* tachyzoites provided substantial, although incomplete, protection against acute challenge with the three *T. gondii* strains evaluated in this study, and protective efficacy against tachyzoite challenge was maintained for up to 75 days after immunization. Following oral challenge with 10 or 40 Pru cysts, brain cyst burdens in surviving RHΔ*rop64*-immunized mice were reduced to 28 ± 44 and 5 ± 13 cysts, respectively, which is broadly comparable to reductions reported for other recently described live-attenuated vaccine strains. Although several vaccine candidates, such as Δ*rop18*, Δ*gra72*, and Δ*cdpk3*, have reported protective immunity lasting up to 120–125 days after vaccination, direct comparisons among studies should be interpreted cautiously because parasite backgrounds, mouse strains, immunization doses, challenge protocols, and evaluation time points differed substantially. Collectively, these findings indicate that RHΔ*rop64* exhibits protective efficacy broadly comparable to recently reported CRISPR/Cas9-generated live-attenuated vaccine strains and further expands the repertoire of genetically attenuated *T. gondii* vaccine candidates.

ROPs have emerged as important determinants of *T. gondii* virulence and promising targets for vaccine development. Many ROP family members, such as ROP18, ROP5, ROP17, and ROP55, play key roles in host–parasite interactions. These proteins contribute to parasite virulence through diverse mechanisms, including modulation of host immune responses and promotion of intracellular parasite survival. Among them, some have been shown to induce protective immune responses when used as vaccine antigens [18,34,35,36,37]. Although the precise molecular function of ROP64 remains unknown, the markedly attenuated virulence of RHΔ*rop64* observed in the present study suggests that ROP64, like several other rhoptry proteins, contributes to parasite virulence and host–parasite interactions. These findings provide an additional rationale for exploring novel ROPs as candidates for vaccine development.

In the present study, deletion of ROP64 significantly attenuated parasite virulence in vivo, as evidenced by the survival of mice infected with RHΔ*rop64* tachyzoites at doses that are lethal for the parental RH strain. This attenuation supports the potential of RHΔ*rop64* as a live attenuated vaccine candidate. Importantly, RHΔ*rop6*4 vaccination conferred substantial, although incomplete, protection against acute challenge with the three *T. gondii* strains evaluated in this study, demonstrating protection across the three *T. gondii* strains evaluated under the present experimental conditions.

In addition to protection against acute infection, RHΔ*rop64* vaccination was associated with reduced brain cyst burdens among surviving mice following chronic challenge. However, because brain cyst quantification was performed only in surviving animals, mice that succumbed to infection before the scheduled sampling time point could not be evaluated. Therefore, these findings should be interpreted with caution, as survival bias may have influenced the comparison of brain cyst burdens between groups. Furthermore, PCR-positive but microscopy-negative results may reflect residual parasite DNA, low-level persistent infection below the detection limit of microscopy, or the persistence of non-viable parasites. Since PCR detects parasite DNA but cannot determine parasite viability, the impact of RHΔ*rop64* vaccination on parasite persistence requires further investigation, and PCR positivity alone should not be interpreted as evidence of parasite viability or sterile immunity.

Immunological analysis further demonstrated that RHΔ*rop64* vaccination induced both humoral and cellular immune responses. Increased levels of total IgG, IgG1, and IgG2a were observed, with IgG2a predominating, resulting in an antibody pattern consistent with a Th1-biased immune profile. In parallel, increased production of IL-2, IL-12, and IFN-γ was detected, together with moderate increases in IL-4 and IL-10. These findings are consistent with previous studies demonstrating that Th1-type immunity plays a central role in protection against *T. gondii* infection [11,29,33,38,39,40]. However, because immune cell subsets and antigen-specific cellular responses were not evaluated in the present study, the underlying cellular mechanisms responsible for these immune responses remain to be elucidated.

The concurrent induction of IL-4 and IL-10 may also contribute to immune regulation by limiting excessive inflammatory responses. Balanced immune responses are essential for host survival, as excessive Th1 responses can lead to immunopathology [41]. Therefore, RHΔ*rop64* immunization was associated with the induction of both pro-inflammatory and regulatory immune responses. Further studies are required to determine the precise immunological mechanisms underlying the protection observed following challenge.

Despite the protective effects observed in the mouse model, several limitations should be considered. Although RHΔ*rop64* exhibited markedly reduced virulence in mice, histopathological analyses and long-term safety evaluations were not performed in the present study. Therefore, additional studies are required to further assess its biological safety in vivo. In addition, the genetic stability of RHΔ*rop64* during in vivo passage and the potential for reversion to virulence were not evaluated in the present study. These aspects should be carefully assessed before practical application of this live attenuated vaccine candidate. Furthermore, whether RHΔ*rop64* is completely cleared or persists in host tissues following immunization remains unknown. Since parasite burden and tissue distribution were not evaluated prior to challenge, the fate of RHΔ*rop64* in immunized animals requires further investigation. The safety of RHΔ*rop64* under immunosuppressive conditions also remains unknown and warrants further investigation. Although protective efficacy was evaluated for up to 75 days after immunization in the present study, longer follow-up periods are needed to better define the durability of vaccine-induced immunity. Moreover, its resistance to pyrimethamine may restrict its application in food-producing animals and preclude its use in humans. In addition, the present study did not evaluate vaccine efficacy in congenital infection models or in infection initiated via oocyst exposure. Future studies should address these limitations and further evaluate the efficacy of RHΔ*rop64* against genetically diverse *T. gondii* strains, including atypical strains circulating in South America, as well as its practical applicability in additional animal models.

## 5. Conclusions

Collectively, these findings suggest that RHΔ*rop64* has the potential to serve as a live attenuated vaccine candidate. Under the experimental conditions evaluated in this study, RHΔ*rop64* provided substantial, although incomplete, protection against acute challenge with the three *T. gondii* strains tested and was associated with reduced brain cyst burdens among surviving mice following chronic challenge. However, this study was conducted only in a mouse model, and further studies are required to comprehensively evaluate its safety, long-term protective efficacy, and applicability in food-producing animals before its potential as a live vaccine candidate can be established. Moreover, protection against the highly virulent RH strain remained incomplete, and chronic infection was not completely prevented. Future studies should therefore focus on improving protective efficacy while further evaluating the safety and long-term performance of attenuated live vaccine candidates.

## Data Availability

The original contributions presented in this study are included in the article/Supplementary Material. Further inquiries can be directed to the corresponding authors.

## Supplementary Materials

The following supporting information can be downloaded at: https://www.mdpi.com/article/10.3390/microorganisms14081725/s1, Supplementary File S1: Table S1. Conventional PCR reaction mixture for *B1* gene detection. Table S2. Conventional PCR cycling conditions for *B1* gene detection. Table S3. Primers used for amplification of the *B1* gene. Supplementary File S2: Table S4. Brain cyst burden, brain cyst-positive mice, and individual brain cyst counts in RHΔ*rop64*-immunized mice challenged with the type II *Toxoplasma gondii* Pru strain (tachyzoites or cysts). Supplementary File S3: Figure S1. Detection of the *T. gondii B1* gene by PCR in brain tissues from surviving RHΔ*rop64*-immunized mice without microscopically detectable brain cysts after challenge. Brain tissues from surviving mice without microscopically detectable cysts were analyzed by PCR targeting the *B1* gene. Only these samples were subjected to PCR analysis. Electrophoresis results are shown for mice challenged with 5 × 10^4^ Pru tachyzoites at 30 dpv (A1–A3) or 75 dpv (B1–B4); with 10 cysts at 30 dpv (C1–C7) or 75 dpv (E1–E5); and with 40 cysts at 30 dpv (D1–D8) or 75 dpv (F1–F7). P, positive control; N, negative control. Reference [42] is cited in the supplementary materials.
