# Supplementary material for: ROP64-Deficient Toxoplasma gondii Strain Confers Protection Against Acute and Chronic Toxoplasmosis in Mice
Source: Microorganisms. 2026 Aug 6;14(8):1725. doi: 10.3390/microorganisms14081725 (PMC13515172; doi:10.3390/microorganisms14081725)
Supplement: Supplementary file 1 [file microorganisms-14-01725-s001.zip › Supplementary file S1 Table S1-S3.pdf]

Conventional PCR targeting the *Toxoplasma gondii B1* gene was performed to assess the presence of parasite DNA in brain tissues from mice without microscopically detectable cysts. The primer sequences used for *B1* gene amplification were adopted from Burg et al. (1989). The PCR reaction mixture, cycling conditions, and primer sequences used in the present study are provided in Tables S1–S3. Following PCR amplification, the products were analyzed by 2% agarose gel electrophoresis at 120 V and visualized using a gel imaging system.

Supplementary file S1: Table S1 Conventional PCR reaction mixture for *B1* gene detection.

**Component**	**Volume/μL**
ddH_2O	16.8
10×PCR buffer (Mg^{2+} free)	2.5
$MgCl_2$	2.0
dNTP	0.5
F1	0.5
R1	0.5
rTaq	0.2
Template DNA	2
Total volume	25

Supplementary file S1: Table S2 Conventional PCR cycling conditions for *B1* gene detection

**Temperature/°C**	**Time**	**Cycle number**
94	5 min	
94	10 s	×30
57	10 s	×30
72	30 s	×30
72	5 min	
16	∞	

Supplementary file S1: Table S3 Primers used for amplification of the *B1* gene

**Primer name**	**Primer sequence (5'→3')**
F1	GGAACTGCATCCGTTCATGAG
R1	TCTTTAAAGCGTTCGTGGTC

Reference:

Burg JL, Grover CM, Pouletty P, Boothroyd JC. Direct and sensitive detection of a pathogenic protozoan, *Toxoplasma gondii*, by polymerase chain reaction. *J Clin Microbiol*. 1989, 27(8):1787-92.
