# Supplementary material for: ROP64-Deficient Toxoplasma gondii Strain Confers Protection Against Acute and Chronic Toxoplasmosis in Mice
Source: Microorganisms. 2026 Aug 6;14(8):1725. doi: 10.3390/microorganisms14081725 (PMC13515172; doi:10.3390/microorganisms14081725)
Supplement: Supplementary file 1 [file microorganisms-14-01725-s001.zip › Supplementary file S2 Table S4.pdf]

Supplementary file S2: Table S4. Brain cyst burden, brain cyst-positive mice, and individual brain cyst counts in RHΔ*rop64*-immunized mice challenged with the type II *Toxoplasma gondii* Pru strain (tachyzoites or cysts)

Group	Mean brain cyst burden	Brain cyst-positive mice, n/N (%)	Individual brain cyst counts (PCR)
Immunized + Pru tachyzoites (30 d)	19 ± 38	1/4 (25%)	75, 0(+), 0(+), 0(-)
Immunized + Pru tachyzoites (75 d)	0	0/4 (0%)	0(+), 0(+), 0(-), 0(+)
Naive +10 cysts (30 d)	4781 ± 5967	4/4 (100%)	13575, 1425, 3375, 750
Immunized +10 cysts (30 d)	19 ± 53	1/8 (12.5%)	150, 0(-), 0(-), 0(-), 0(-), 0(+), 0(+), 0(+)
Naive +40 cysts (30 d)	NA	NA	No surviving mice
Immunized + 40 cysts (30 d)	0	0/8 (0%)	0(+), 0(+), 0(+), 0(+), 0(+), 0(-), 0(-), 0(-)
Naive +10 cysts (75 d)	750	1/1 (100%)	750
Immunized +10 cysts (75 d)	28 ± 44	3/8 (37.5%)	113, 38, 75, 0(+), 0(+), 0(+), 0(-), 0(-)
Naive +40 cysts (75 d)	1613	1/1 (100%)	1613
Immunized + 40 cysts (75 d)	5 ± 13	1/8 (12.5%)	38, 0(+), 0(+), 0(+), 0(+), 0(+), 0(+), 0(-)

Abbreviations: NA, not applicable because no mice survived to the scheduled sampling time point.

Mean brain cyst burden was calculated using all surviving mice, including mice with zero microscopically detectable brain cysts. For groups with only one surviving mouse, the individual cyst count is presented without calculation of the standard deviation.

Brain cyst-positive mice represent mice with microscopically detectable brain cysts and are presented as n/N (%), where N represents the number of surviving mice available for brain cyst examination in each group, whereas n represents the number of mice containing brain cysts among the surviving mice. PCR analysis was performed only on brain tissues from surviving mice without microscopically detectable brain cysts.

Values in the **"Individual brain cyst counts (PCR)"** column represent individual brain cyst counts. Values shown as 0(+) indicate that no brain cysts were detected by microscopy, whereas the corresponding brain tissue was positive for the *T. gondii B1* gene by PCR. Values shown as 0(−) indicate negative results by both microscopy and PCR. "No surviving mice" indicates that no animals survived to the scheduled sampling time point.

PCR positivity indicates the presence of *T. gondii* DNA but does not distinguish between viable and non-viable parasites.
